# The International Medical Congress

**Published:** 1887-09

**Authors:** 


THE INTERNATIONAL MEDICAL CONGRESS.—The Ninth Annual Session of this Congress now in session in Washington City is largely attended, and the meetings have proven very interesting and instructive. The meetings of the Dental Section have been very well attended, and the clinics of a high order. *The Medical Record*, Wm. Wood & Co., publishers, are to be commended for their generosity and kindness in supplying all the medical and dental journals of the country with proof sheets of the proceedings which will be appreciated by all who receive them as furnishing an excellent account of the proceedings of this distinguished body of gentlemen. In the next number of the JOURNAL we will furnish our readers with the proceedings of the Dental Section.

